# Atypical and severe manifestations of chikungunya virus infection in French Guiana: A hospital-based study

**DOI:** 10.1371/journal.pone.0207406

**Published:** 2018-12-06

**Authors:** Timothee Bonifay, Christelle Prince, Clarisse Neyra, Magalie Demar, Dominique Rousset, Hatem Kallel, Mathieu Nacher, Félix Djossou, Loïc Epelboin, and the Char Chik Working group

**Affiliations:** 1 Tropical and Infectious Diseases Department, Centre Hospitalier Andrée Rosemon, Cayenne, French Guiana; 2 Department of General Medicine, University of the French West Indies, Pointe-à-Pitre, Guadeloupe; 3 Centre d’Investigation Clinique Antilles Guyane, Inserm CIC 1424, Cayenne, French Guiana; 4 Laboratoire Hospitalier universitaire d'immunologie, Centre Hospitalier Andrée Rosemon, Cayenne, French Guiana; 5 Equipe EA 3593, Ecosystèmes Amazoniens et Pathologie Tropicale, Université de Guyane, Cayenne, French Guiana; 6 National Reference Center for arboviruses, Institut Pasteur de la Guyane, Cayenne, French Guiana; 7 Intensive Care Unit, Centre Hospitalier Andrée Rosemon, Cayenne, French Guiana; Agency for Science, Technology and Research - Singapore Immunology Network, SINGAPORE

## Abstract

**Background:**

French Guiana (FG) was the first country in South America to declare chikungunya virus infection (CHIKV). The outbreak affected about 16,000 persons between February 2014 and October 2015, with several atypical cases, but only two fatal cases. We aimed to describe the clinical presentation of patients hospitalized for CHIKV infection, to estimate and identify risk factors of unusual and severe forms in adult patients.

**Materials and methods:**

A monocentric retrospective study was conducted in Cayenne hospital, the main city and the main hospital in FG, from March 1^st^ 2014 to August 31^st^ 2015. All patients admitted for at least one night with a biological diagnosis of CHIKV infection during the 2014/2015 outbreak were included, except pregnant women and children under 15 years.

**Results:**

During the study period, 285 patients with a diagnosis of CHIKV infection were hospitalized in Cayenne hospital, among whom 96 nonpregnant adults were studied. Five were classified as severe forms (5.2%) and 23 as unusual forms (23.9%). The most frequent atypical and/or severe form was neurological (n = 20), followed by cardio-respiratory failure (acute respiratory failure n = 4, acute heart failure n = 2), digestive and hepatic disorders (acute hepatitis n = 3, acute pancreatitis n = 2), renal disorders (acute renal failure n = 5) and muscular impairment (rhabdomyolysis n = 3).

**Conclusion:**

During the outbreak, hospitalizations were frequent, particularly for common forms, driven by algic clinical presentations and concerns due to the novelty of this infection. Despite atypical neurological and liver forms of CHIKV infection, case-fatality was low in French Guiana. No specific risk factor of atypical and/or severe forms was found in our study.

## Introduction

Chikungunya virus (CHIKV) is an arthropod-borne ARN virus belonging to the Alphavirus genus of the family of Togaviridae that is transmitted by *Aedes* sp. mosquitoes. It was first isolated in 1952 in Tanzania but was never really considered as a virus of interest, despite several outbreaks in Asia and Africa, before the Indian Ocean outbreak in 2005–2006 [1–3]. Until this huge outbreak, clinical manifestations were considered as mild and self-limited in evolution, but with persistent joint pains. However, in 2005 chikungunya fever unexpectedly re-emerged in the form of devastating epidemics in the Indian Ocean islands [4]. The outbreak was particularly well described on La Reunion Island where one third of the population was suspected to have been infected between December 2005 and June 2006 [5]. Beside common forms, atypical and severe forms due to CHIKV were described for the first time. Knowledge acquired during this period allowed a better understanding of the pathology. Most of the initially labeled “atypical forms” were then considered as common during the following epidemics. Besides exacerbations of previous chronic underlying diseases, several CHIKV specific manifestations were reported: neurological (encephalitis, Guillain-Barré syndrome), cardiac (myocarditis, pericarditis), acute hepatitis and acute renal failure [4,6–9].

In December 2013, an outbreak of CHIKV started from the French Caribbean, and then spread to all the Caribbean and Latin America. French Guiana, a French overseas territory located on the Northeastern coast of South America, was the first area on the continent to report cases, presumably because of the strong links and numerous travels between the French Caribbean and French Guiana. The epidemic affected about 16,000 persons between February 2014 and October 2015, with several atypical cases, but only two fatal cases [10,11]. Although atypical and severe cases have been widely reported from La Reunion Island, few clinical studies have been published reporting the Latin American experience [12–15]. Thus, we aimed to describe the clinical presentation of patients hospitalized for CHIKV infection in French Guiana, to estimate and identify risk factors of unusual and severe forms in adult patients.

## Material and methods

### Study design and population

A monocentric retrospective and descriptive study was conducted in Cayenne hospital, the main hospital in FG, analyzing data collected from March 1^st^ 2014 to August 31^st^ 2015. All patients admitted for at least one night with a diagnosis of CHIKV infection during the 2014/2015 outbreak were included.

### Setting

French Guiana is a French overseas territory located between Brazil and Surinam with an estimated population of 256 000 inhabitants. Because of its geographical location, it is exposed to tropical pathologies while benefiting from health facilities with European standards.

### Inclusion criteria and case definition

A case of CHIKV infection was defined as a compatible clinical presentation and a positive microbiological diagnosis of acute infection by CHIKV, by positive RT-PCR or positive anti CHIKV IgM antibodies. Nucleic acids were extracted from serum using a MagnaPure LC total nucleic acid isolation kit and a MagnaPure LC 2.0 instrument (Roche). Real time RT-PCR amplification was realized with the RealStar Chikungunya RT-PCR kit 1.0 CE-IVD marked (Altona Diagnostics). CHIKV serodiagnosis were performed by the National Reference Center (NRC) for arboviruses in French Guiana in the Institute Pasteur de la Guyane, with anti CHIKV IgM antibodies detection by an in-house MAC-ELISA test based on whole virus-based antigens and hyperimmune ascitic fluid.

Each case was then screened by two independent experts to classify the patient into the common, atypical or severe form. The definition of these forms relied on a definition by infectious diseases experts from the French Caribbean and French Guiana, issued from the experience of La Reunion Island [16]: (1) A common form was defined as a CHIKV infection with fever (defined as body temperature > 38°C) or sensation of fever, and/or joint paint or arthritis, with one of several classical signs: joint edema, synovitis, pruritus, macular or papular exanthema, nausea, vomiting, diarrhea or abdominal pain. (2) An atypical form was defined as one of the following cases: hyperalgic syndrome (requiring the use of corticosteroid and/or morphine), neurological disorder such as encephalitis, encephalopathy (Glasgow > 8) or stroke, cardiac disorder (with or without preexisting cardio-respiratory disease), unusual dermatological anomaly, respiratory disorder (requiring at least for 2 l/min oxygen), acute kidney injury (defined as KDIGO stage 2), liver function disorder (defined as ALT or AST >10 times the baseline value), exacerbation of preexisting chronic disease, rhabdomyolysis (CPK > 5 times the baseline value [17]), minor hemorrhagic syndrome or thrombotic complications. (3) A severe form was defined as cardio-circulatory, neurological, respiratory, renal or hepatic failure.

### Exclusion criteria

The exclusion criteria were an age under 15 years and ongoing pregnancy. Children and pregnant women were monitored in the departments of pediatrics and gynecology-obstetrics, respectively, and thus did not allow standardized data collection. Furthermore, pregnant women were generally admitted for pregnancy monitoring and to avoid premature deliveries without procuring forms of CHIKV infection. However, although they were not included in the analysis of clinical presentations, they were included for the calculation of length and cost of hospitalization.

### Data collection and analysis

The data were retrospectively collected. Data included demographic information (gender, age), preexisting co morbidities, which were summarized by using the Charlson index [18], which predicts 10-year survival in patients with multiple comorbidities, clinical manifestations described by organ system, biological analysis and data related to hospitalization, admissions in intensive care and/or other intermediate and step-down care units and events that occurred during hospitalization. The first day of fever was defined as the first day of symptoms. To estimate the costs associated with CHIKV infection, the initial and subsequent hospitalization costs were calculated using the 2014 official French diagnosis related group (DRG) pricing for public hospitalizations. In order to estimate total costs, the computations used the number of hospitalization days for each ward. To enhance the identification of risk factors for atypical or severe cases, we performed two comparisons: severe + atypical vs. classical and severe vs. atypical + classical. The epidemiological characteristics of the groups were compared using a chi-square test for qualitative variables, a Student test for the normally distributed quantitative variables, or a non-parametric Mann and Whitney test for the other continuous variables. Fisher test was used to analyze small group. Differences were considered significant if the p value was <0.05. Analyses were performed with Stata software, version 12.0.

### Ethics

The present study was monocentric, retrospective and consisted of anonymized patient records (the database did not include names or any variable that could allow the precise identification of patients) as authorized by the French regulatory authorities. The database was declared to the Commission National Informatique et des Libertés (CNIL N°TFN1490159N) following French legal requirements.

## Results

### General population

**Fig 1:**
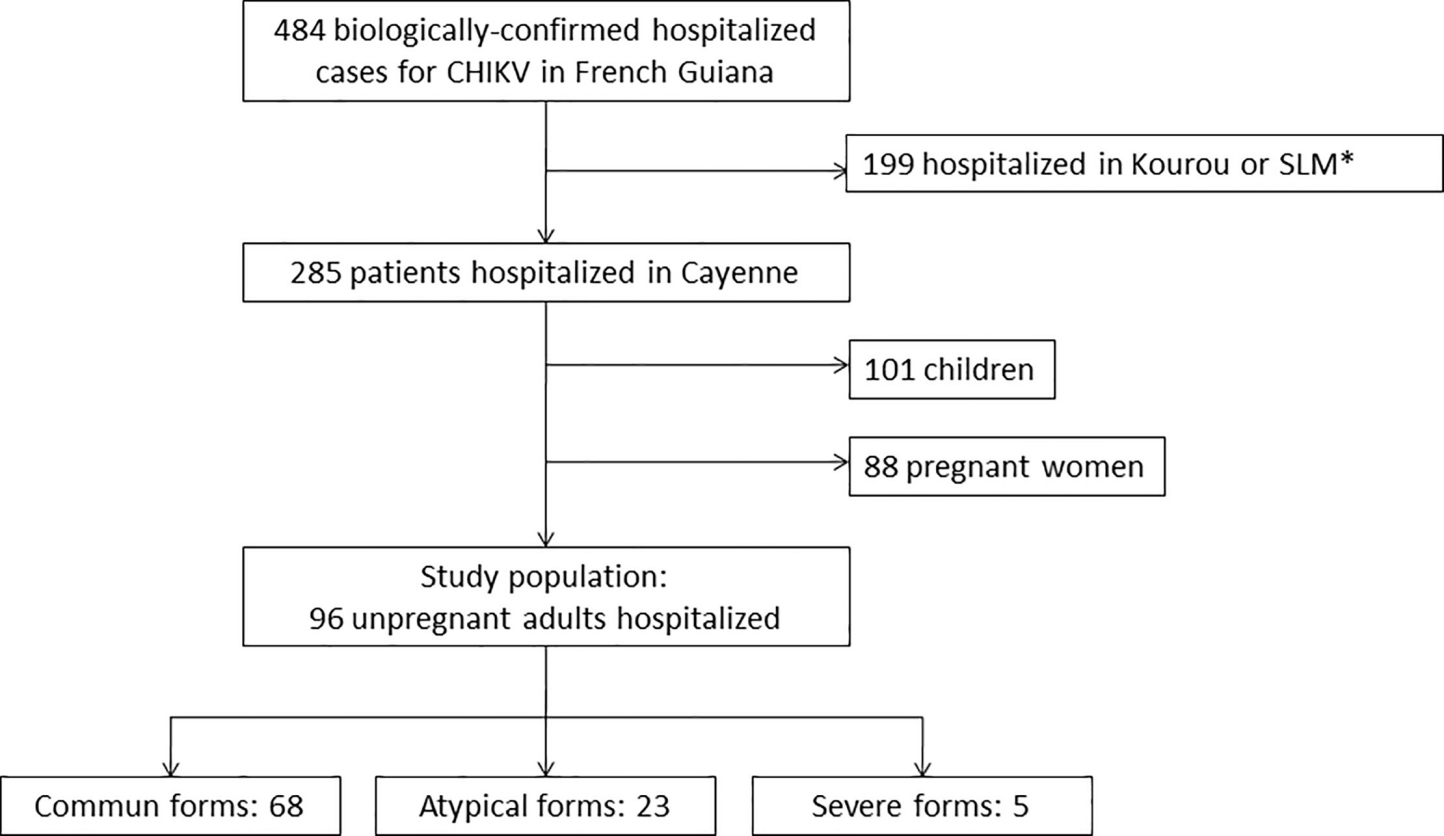
Study flow chart. *SLM: Saint Laurent du Maroni.

During the study period, 285 patients with a diagnosis of CHIKV infection were admitted for more than one night at Cayenne hospital (Fig 1). Among them 101 children under 15 years and 88 pregnant women were hospitalized but not included in the analysis. Among the 96 non-pregnant adults included in the study, 68 (71%), 23 (24%) and 5 (5%) were classified as common, atypical or severe forms, respectively. The median age of patients was 57 years (IQ 41–69.5; range 17.3–98.3) and 49 patients (51.0%) were female (Sex ratio M/F = 0.96). Sixty-nine (72%) patients had a preexisting condition; the most frequent were high blood pressure (31%), diabetes mellitus (25%), rheumatic diseases (14%), chronic kidney disease (12%) and HIV infection (12%). The median average Charlson score was 2 (IQ 0–4) and 32 patients (33%) had more than one comorbidity. The median duration of hospitalization was 5 days (IQ 3–8; range 1–136). Eighty-six patients (90%) were diagnosed by PCR and 10 (10%) by anti-CHIK IgM. Among cases (N = 96), 94.8% had fever and 71.9% presented arthralgia. Other reported symptoms were headache (38.5%), myalgia (33.3%), back pain (32.3%), nausea (24%), rash (14.6%), abdominal pain (14.4%), diarrhea (12.5%) and joint edema (11.5%). The most frequent atypical and/or severe forms were neurological (n = 12) with Guillain-Barré syndrome n = 2, encephalitis n = 3, seizure n = 2, confusion n = 4, and stroke n = 1. It was followed by cardio-respiratory failure (acute respiratory failure n = 4, acute heart failure n = 2), digestive and hepatic disorders (acute hepatitis n = 3, acute pancreatitis n = 2), renal disorders (acute renal failure n = 5) and muscular impairment (rhabdomyolysis n = 3). The remaining atypical forms were an atypical case of parotiditis, sepsis, hydrocholecystis and a need of high doses of corticosteroid therapy (Table 1). Nine (10.5%) patients had an associated bacterial infection (3 with respiratory tract infection and 6 with urinary tract infection).

**Table 1:** Atypical and severe cases description.

Case	Sex	Age (yo)	Medical story	Associated bacterial infection	Details of atypical and severe forms
**Severe 1**	F	42	-	-	Thrombotic thrombocytopenic purpura
**Severe 2**	M	31	-	-	GBS ARespF
**Severe 3**	M	57	-	-	ARF (dialysis) acute hepatitis
**Severe 4**	M	55	Chronic pancreatitis, OH	-	Acute pancreatitis, GBS, encephalitis, temporo-spatial disorientation
**Severe 5**	M	49	Dm	-	Acute hepatitis
**Atypical 01**	M	19	-	-	Severe sepsis
**Atypical 02**	M	63	HBP	-	Temporo-spatial disorientation
**Atypical 03**	M	67	HBP	-	Encephalitis, Temporo-spatial disorientation
**Atypical 04**	F	73	Sarcoidosis	UTI	ARF
**Atypical 05**	M	80	CRF,Dm, HBP, stroke	-	Rhabdomyolysis
**Atypical 06**	F	62	Cancer Dm	-	Exacerbation of cirrhosis, temporo-spatial disorientation
**Atypical 07**	M	68	HBP	-	Stroke, temporo-spatial disorientation
**Atypical 08**	F	85	HBP, stroke	-	Acute heart failure
**Atypical 09**	M	74	HBP, stroke	-	ARF, rhabdomyolysis
**Atypical 10**	F	31	Hydrocephaly	-	Seizure, temporo-spatial disorientation
**Atypical 11**	M	79	CRF,Dm, HBP, PAD, stroke	-	ARespF
**Atypical 12**	F	73	Dm, HBP, PAD, stroke	RTI	Seizure, temporo-spatial disorientation
**Atypical 13**	F	28	SCD, Crohn’s disease	-	Temporo-spatial disorientation, cholecystitis
**Atypical 14**	F	70	HIV	-	Encephalitis, rhabdomyolysis, temporo-spatial disorientation
**Atypical 15**	M	54	chronic pancreatitis, Dm, OH	-	Acute pancreatitis and hepatitis
**Atypical 16**	F	68	-	-	Paroditis
**Atypical 17**	M	58	CKD CRF HIV	RTI	ARF, ArespF
**Atypical 18**	F	38	Immunosuppression none HIV	-	Hyperalgic syndrome
**Atypical 19**	M	69	HBP	-	Acute heart failure
**Atypical 20**	M	58	Dm, stroke	-	Temporo-spatial disorientation
**Atypical 21**	F	41	Cancer, HIV, CResF	RTI	ARespF (exacerbation CResF)
**Atypical 22**	M	71	Dm, HBP, Cancer	-	Encephalitis, temporo-spatial disorientation
**Atypical 23**	F	31	-	UTI	ARF

ARespF Acute respiratory failure; ARF acute renal failure; CKD chronic kidney disease; CResF Chronic respiratory failure; CRF chronic respiratory failure; Dm diabete mellitus; GBS Guillain-Barré syndrome; HBP high blood pressure; HIV Human Immunodeficiency Virus; OH chronic alcoholism; PAD peripheric arterial disease; RTI respiratory tract infection; SCD sickle cell disease; UTI urinary tract infection.

### Severe forms

Five patients had a severe CHIKV infection, according to our classification. One case, already reported [11], was fatal following a thrombotic thrombocytopenic purpura two patients developed Guillain-Barré syndrome and two patients underwent acute hepatitis with cytolysis above ten times normal range without liver failure, among them one associated rhabdomyolysis and a second an acute renal failure requiring emergency dialysis. Another fatal case reported in French Guiana was linked to CHIKV infection but was not hospitalized and died at home, so not included in our study. CHIKV infection was notified on the death certificate [10]. One patient presented severe sepsis without any other microbiological documentation with a quick improving and was classified atypical form of CHIKV infection.

### Risk factors of atypical and/or severe cases

Bivariate analysis revealed no difference in clinical or biological presentation. A delayed consultation and a delayed diagnosis appeared to be in favor of severe or unusual form of CHIKV infection (Table 2). Charlson score and age > 60 years tended to be more frequent in patients with atypical and severe forms vs. common forms, but were not significant.

**Table 2:** Bivariate analysis of epidemiological, clinical and biological data by severity classification.

variable	Common n = 68 (%)	Atypical n = 23(%)	Severe n = 5 (%)	Commune vs Atypical & severe		Commune & atypical vs severe	
				OR [CI 95%]	p	OR [CI 95%]	p
**Age > 60 yo**	28 (41.2)	13 (56.5)	0 (0)	-	0.64	-	0.06
**Low income area**	45/60 (75.0)	15/20 (75.0)	2/2 (100)	-	0.83	-	0.082
**Born in the foreign**	42 (61.8)	13/21 (61.9)	4 (80.0)	-	0.75	-	0.68
**Medical history**	47 (69.1)	20 (87.0)	2 (40.0)	-	0.35	-	0.13
**Previous rhumatismal diseases**	9 (13.2)	4 (17.4)	0 (0)	-	0.89	-	0.48
**Charlson> 2**	26 (38.2)	13 (56.5)	0 (0)	-	0.46	-	0.07
**Headache**	29 (42.7)	6 (26.1)	2 (40.0)	-	0.20	-	0.64
**Muscle pain**	24 (35.3)	6 (26.1)	2 (40.0)	-	0.53	-	0.54
**Joint pain**	54 (79.4)	13 (56.5)	2 (40.0)	0.30 [0.12–0.76]	0.01	-	0.13
**Diarrhea**	8 (11.8)	4 (17.4)	0 (0)	-	0.73	-	0.50
**Vomiting**	10 (14.7)	6 (26.1)	2 (40.0)	-	0.11	-	0.24
**Abdominal pain**	10 (14.7)	3 (13.1)	1 (20.0)	-	0.96	-	0.55
**Back pain**	26 (38.2)	4 (17.4)	1 (20.0)	0.35 [0.09–1.12]	0.05	-	0.48
**Arthritis**	8 (11.8)	3 (13.0)	0 (0)	-	0.88	-	0.54
**Hb< 12 g/dL**	17 (25.8)	6 (26.1)	3 (60.0)	-	0.53	-	0.17
**WBC> 10/mm3**	7 (10.3)	4 (17.4)	1 (20.0)	-	0.31	-	0.50
**PMN > 8/mm3**	7 (10.3)	3 (13.0)	1 (20.0)	-	0.58	-	0.46
**Lymphocytes< 0.5/mm3**	12 (17.7)	2 (8.7)	0 (0)	-	0.19	-	0.45
**Platelet count< 150G/L**	13 (19.1)	3 (13.0)	1 (20.0)	-	0.57	-	0.63
**CRP > 100**	8 (12.3)	2 (9.1)	1 (25.0)	-	0.92	-	0.19
**More than 5 days of** **hospitalization**	23/65 (35.4)	11 (47.8)	5 (100.0)	-	0.05	0 [1.98 - ∞]	0.02
**Delayed diagnosis (more than 2 days after beginning of signs)**	21 (30.9)	10 (43.5)	5 (100)	-	0.48	0 [1.52 - ∞]	0.02

Hb: hemoglobin; WBC: White blood cell; PMN: polymorphonuclear neutrophil; CRP: protein C system; OR: Odds Ratio; CI: Confidence interval

### Hospital acquired infections

Six cases (6.3%) were acquired in the hospital in patients admitted for another cause, among whom 3 were hospitalized in a nursing home, 2 in lost to follow-up patients living with HIV and one for colon cancer. Infection occurred between day 15 and 25 during hospitalization. The last patient was diagnosed CHIKV positive in retrospect by PCR on spinal fluid 23 days after the beginning of the symptoms. Five were classified common form and one atypical. All of them had co morbidities with a Charlson score between 5 to 10.

### HIV population

Among the 96 patients, 11 (11.5%) were living with HIV. The median age was 43 years (IQ 36–59). Of the 11 patients, 8 had CD4 less than 300 and 3 had an atypical form of CHIKV infection. Atypical forms were not related to CD4 count or viral load. Two patients developed acute respiratory failure and one developed non-biologically confirmed encephalitis associating confusion and central facial paralysis. None of them presented a severe form.

### Hospital stay characteristics and costs

**Fig 2:**
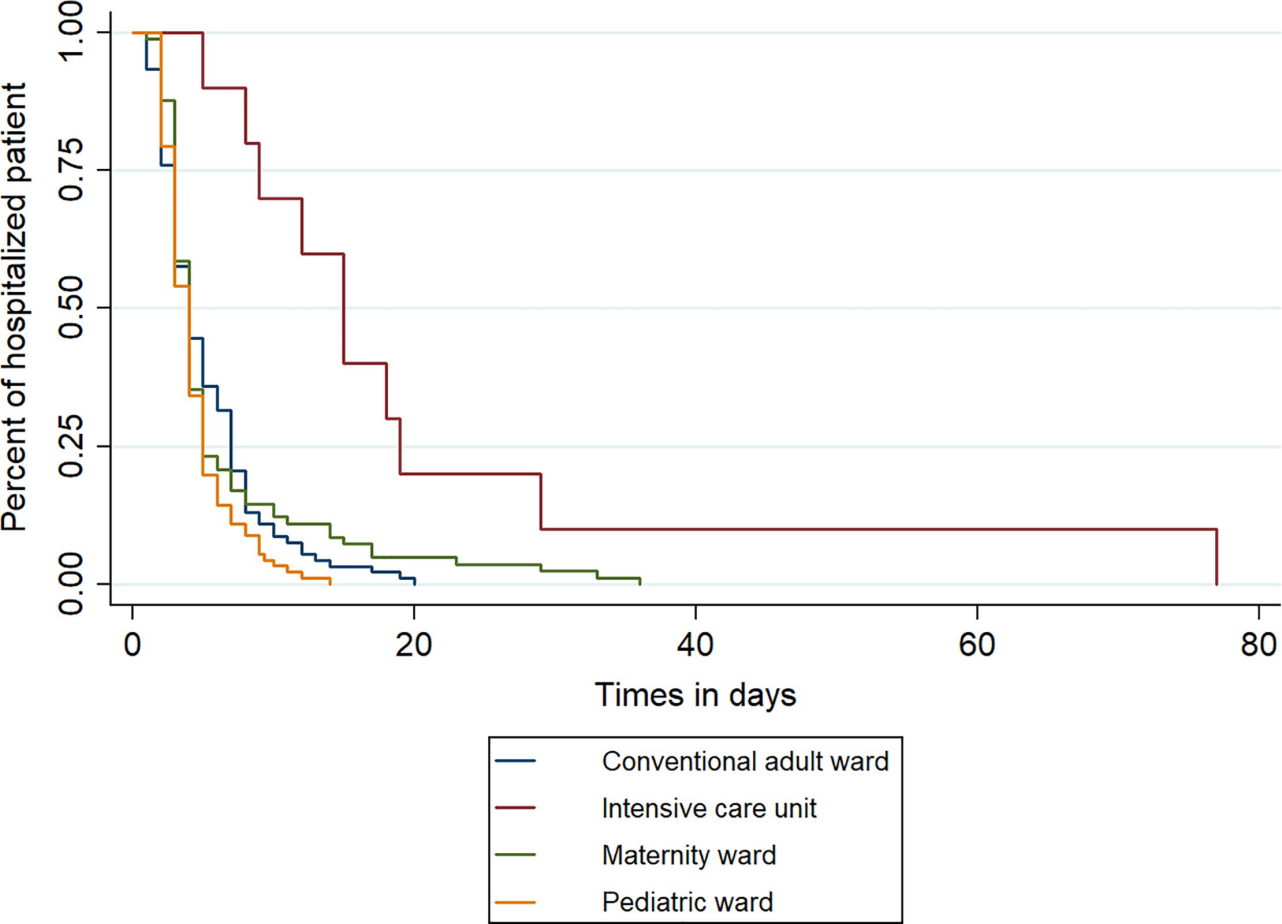
Length of hospitalization estimate by Kaplan Meier by ward.

**Fig 3:**
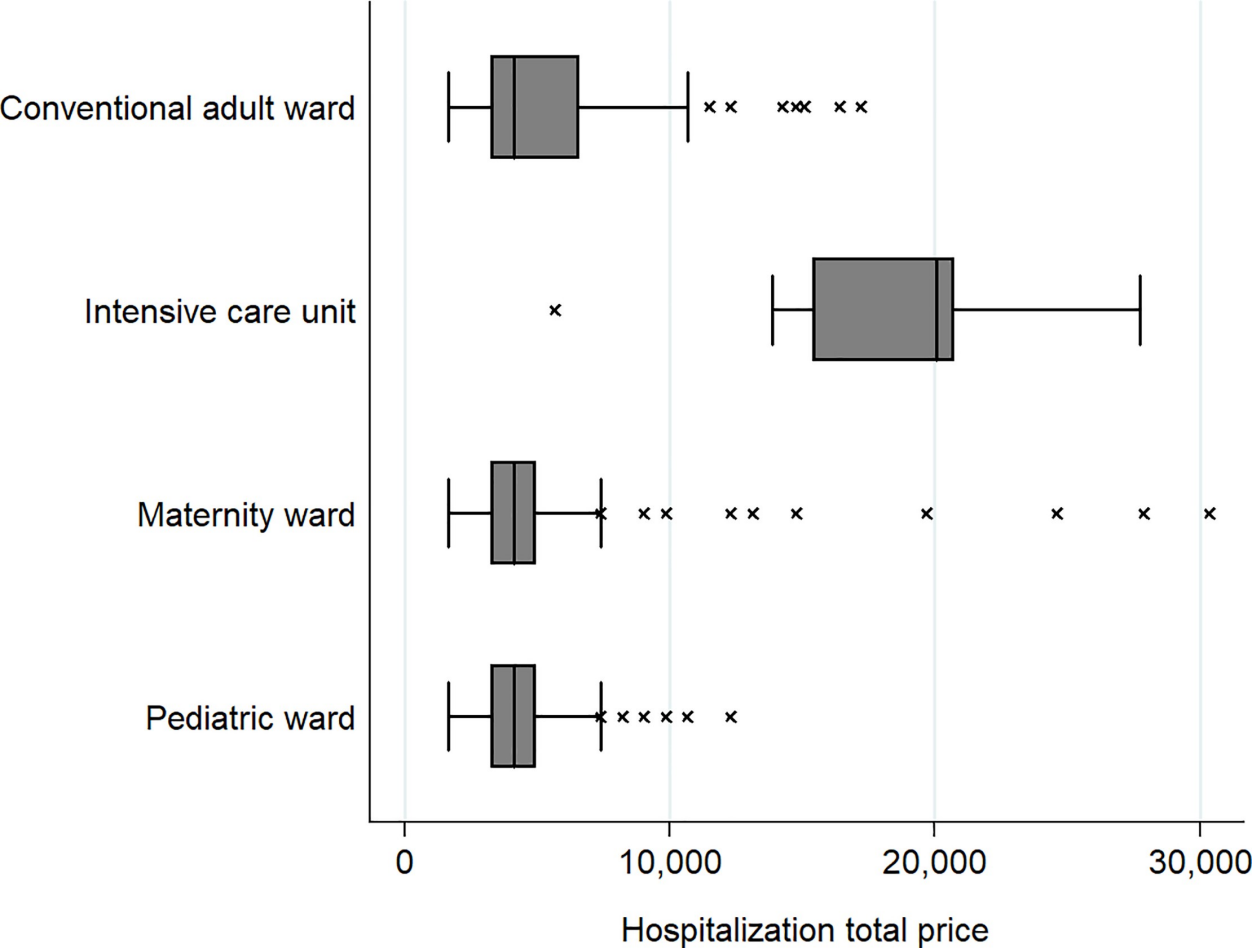
Hospitalization costs according by ward.

Length of stay of hospitalized patients and estimated costs were computed using all 285 patients hospitalized, including children and pregnant women. Average length of stay of patients hospitalized was 6.7 days (SD 6.5; range 2–78). Five patients stayed 30 days or more, among whom 2 in the intensive care unit (Fig 2). Median cost of stay for the French health insurance was € 4,102 (IQR [3,281–5,742]; range 1,640–121,076). Costs of hospital stays differed considerably depending on the ward in which patients were admitted (Fig 3). The median cost of patients who went to the intensive care unit was €14,653 (IQR [10,868–20,052]), while for those in conventional adult, pediatric or maternity services it was €4,102 (IQR [3,281–5,742]). The cost of the Chikungunya outbreak was estimated at € 1,654,137 for 1,862 cumulative hospitalization days.

## Discussion

This hospital-based study described atypical forms which had not been described during the Indian Ocean outbreak. Although the outbreak in La Réunion allowed a better understanding and new recommendations [16,19], in the Americas there have been reports of unknown or unusual forms and a high prevalence of Guillain-Barré syndrome [20,21] or septic shock [12,13,22]. More than two third of hospitalized patients had common forms; three main clinical presentations were mostly reported: total functional impotence because of exhaustion or uncontrolled pain, iterative vomiting and fever resisting to antipyretic treatment. Considering the low number of atypical and severe cases, the number of patients hospitalized for CHIKV was quite high and comparable to the number of DENV patient hospitalized during the 2013 outbreak even if DENV infection is considered as more serious [23–25]. An explanation for the large number of common forms is a better understanding of the disease, most of the atypical forms according to La Reunion classification were then considered as common in 2014 in the Americas (for example rash and abdominal symptoms). Despite these two outbreaks, obtaining uniform classification was difficult, and the implementation of recommendations seemed to be too subjective and clinician-dependent. The proportion of patients with preexisting comorbidities was quite high in the present study (72%). Indeed, when the outbreak began in FG, the main available knowledge about this new disease came from the experience in La Reunion island, from where all the studies showed that the patients with comorbidities where at high risk of complication or death. Thus, these patients were readily admitted even in the absence of atypical or severe form. This is sustained by the observation of the same proportion of comorbidities in the 3 groups common, atypical and severe. Case fatality during the outbreak appeared to be much lower than elsewhere, with two deaths for more than 16,000 suspected cases during the study period, whereas other territories in the French West Indies reported a higher case fatality [10]. Guadeloupe reported 75 deaths and Martinique 85 deaths during the same outbreak (census for Guadeloupe, Martinique and French Guiana, respectively, on January, 1^st^ 2014 : 400,187 and 383,910, and 252,338 [26]). The outbreaks in these territories was due to the same strain, from the Asian lineage [27]. La Reunion, despite a wide range of atypical forms,reported 36% of severe forms, which are associated with a 30% mortality, much higher than in French Guiana [6]. This may be explain by lineage difference of CHIKV, a study found a higher rate of chronic forms in Indian Ocean Lineages, but currently no study has compared atypical and severe form by strains [28]. The population of French Guiana is younger than that of the French Caribbean (www.insee.fr) which may also explain such a difference in morbidity and mortality of the CHIKV outbreak [14]. Similar trends are observed for the other arboviruses epidemics, dengue virus and Zika virus, which provided less severe and fatal cases in French Guiana, than in the French Caribbean [29–31]. Even if less atypical forms were notified, the neurological tropism of CHIKV was pointed out, 42.9% of the atypical and severe forms had neurological manifestations and 2 cases of GBS (Table 3). The French West Indies reported 13 cases of GBS directly attributed to CHIKV [20].

**Table 3:** Comparison of different hospital based studies. Studies were focused on atypical and severe forms of CHIKV infection.

Etude	Economopoulou et al	Tandale et al	Rollé et al	Bonifay et al
**Place**	La Réunion	Ahmedabad (India)	Guadeloupe	French Guiana
**Year**	2005/06	2007	2014	2014/15
**Number (atypical** **and severe forms)**	610	65	76	28
**Age > 60 years**	Median 70 yo	50 (77%)	-	13 (46%)
**Comorbidities**	546 (89%)	20 (31%)	-	22 (79%)
**Severe forms**	222 (36%)	-	42 (55%)	5 (18%)
**Death**	65 (11%)	18 (28%)	14 (18%)	1 (4%)
**Neurologic**	**147 (24**%)	**40 (62**%)	**33 (43**%)	**12 (43**%)
GBS	4 (1%)	-	-	2 (7%)
Meningoencephalitis / Encephalitis	84 (14%)	-	-	3 (11%)
Epileptic seizures	12 (2%)	-	-	2 (7%)
Other	2	-	-	5 (18%)
**Renal**	161 (27%)	45 (70%)	32 (42%)	5 (18%)
**Hepatic**	43 (7)	23(35)	13 (17)	3 (11)
**Pancreatitis**	12 (2)	-	-	2 (7)
**Respiratory**	150 (25)	21(32)	38 (50)	4 (14)
**Cardiac**	226 (37)		38 (50)	2 (7)
**Hematologic**				1 (4)

GBS Guillain-Barré syndrome

It is interesting to note that we found almost no specific risk factor for atypical and or/severe forms of Chikungunya infection, especially age and medical history. Indeed, several risk factors for severe forms had been identified during the La Réunion outbreak, such as cardiac or respiratory failure history, history of hypertension, and taking Nonsteroidal anti-inflammatory drugs (NSAIDs) prior to hospitalization. Risk factors for death were alcohol consumption and age > 85 years old [6]. A longer diagnosis delay was associated with severe forms, which may be due to a greater difficulty and delay to make the diagnosis, as other infectious agents were first ruled out. There are no satisfactory explanations why classifiable risk factors were not found in our study. We may simply note that NSAIDs are rarely used in French Guiana, as the population is aware of the risk of severe forms in case of dengue fever, an arbovirus that provides frequent outbreaks in the territory.

During arboviral outbreaks preventive actions are important. Unfortunately, 6% of all hospitalized cases were acquired in the hospital, especially in a nursing home where protective measures like systematic mosquito nets may be difficult to implement. In the infectious and tropical diseases department, patients with suspicion of chikungunya infection were admitted in the same ward as immunosuppressed patients like those with AIDS or cancer. It is noteworthy that most of Cayenne hospital’s medical wards have no air conditioning, and that not all the patients have a mosquito net. Actions must be systematic during outbreaks, every patient must be protected by a mosquito net, wards must be air conditioned, biological diagnosis must be accelerated and vector control optimal particularly in the hospital.

We provided information about hospitalization costs for chikungunya in Cayenne. This cost was estimated to be 1.6 million Euros while hospitalizations were estimated 8.5 million Euros during the chikungunya outbreak of 2005–206 in La Réunion [32]. Using 2015 official census, 260,000 inhabitants in FG vs. 850,000 in La Reunion island, and reported of population, hospitalization cost was 1.6 times more important in La Reunion than in French Guiana. Whether our estimation is really less comprehensive that the latter study, because it doesn’t take all hospital in French Guiana, didn’t take into account total direct medical costs (consultations, serological tests, drug consumption and hospitalization) and indirect medical costs (such as disease-related loss of productivity), it provides interesting information because the cost of hospitalizations is rarely studied for arbovirus especially for chikungunya, and when it is done it is in countries with lower resources such as India [33].

Unfortunately, the study was monocentric and did not include hospitalized patients from other hospitals in French Guiana.

## Conclusions

In conclusion, during the CHIKV outbreak, hospitalizations were frequent, particularly for common forms, presumably motivated by the combination of patients in pain and concerns about a new virus and possibly the context of intense media attention. Despite neurological and liver atypical forms of CHIKV infection, case fatality was low in the general population, but also in atypical and severe forms.
